# FMT reduces systemic inflammatory response in severe acute pancreatitis by increasing the abundance of intestinal bifidobacteria and fecal bacteria

**DOI:** 10.17305/bb.2024.11445

**Published:** 2024-12-31

**Authors:** Yanning Mao, Yandong Huang, Weiwei Zhang, Huiping Liang, Fengming Liu, Qi Luo, Chunqin Xu, Yi Qin, Jiawen Liu, Shaobo Tang, Huaying Liu, Xiaolong Ge

**Affiliations:** 1 Department of Gastroenterology, The First People’s Hospital of Nanning, Nanning, China; 2 Department of Medicine, GuangXi Health Science College, Nanning, China; 3 Critical Care Medicine, The First People’s Hospital of Nanning, Nanning, China; 4 Department of General Surgery, Sir Run Run Shaw Hospital, School of Medicine, Zhejiang University, Hangzhou, China

**Keywords:** Severe acute pancreatitis, SAP, fecal microbial transplantation, FMT, clinical efficacy

## Abstract

Severe acute pancreatitis (SAP) is one of the leading causes of hospital admissions for gastrointestinal diseases, with a rising incidence worldwide. Intestinal microbiota dysbiosis caused by SAP exacerbates systemic inflammatory response syndrome and organ dysfunction. Fecal microbiota transplantation (FMT) has emerged as a promising therapeutic option for gastrointestinal diseases. In this study, fecal samples from healthy, control, and FMT-treated groups were analyzed using 16S rRNA sequencing to assess microbiome abundance and diversity. Composition and functional prediction analyses were conducted to explore the mechanisms underlying FMT in SAP. FMT significantly improved clinical parameters in SAP patients, including leukocyte count, C-reactive protein (CRP), neutrophil granulocyte count, lactate dehydrogenase (LDH), and calcitonin (*P* < 0.05). Organ failure rates significantly increased in the control group but decreased in the FMT group after treatment (*P* < 0.05). Fecal microbiota sequencing revealed that FMT significantly upregulated the abundance of *Bifidobacterium longum* among all SAP patients (*P* < 0.05). Receiver operating characteristic (ROC) curve analysis indicated that *Bifidobacterium longum* might play a critical role in the efficacy of FMT, with an area under the curve (AUC) value of 0.84. Additionally, there was a negative correlation between *Bifidobacterium longum* abundance and procalcitonin (PCT) levels, as well as a negative correlation between *Escherichia coli* abundance and both CT and Ca values (*P* < 0.05). The relative abundances of *Bifidobacterium longum* and *Escherichia coli* were significantly higher in the FMT group compared to the *Bifidobacterium* triple viable group (*P* < 0.05). In conclusion, this research supports FMT as a safe and effective intervention for treating SAP patients.

## Introduction

Acute pancreatitis is a common cause of acute abdominal pain in clinical settings, marked by localized inflammation of the pancreas. Mild cases often resolve within 1–2 weeks. However, severe acute pancreatitis (SAP) can lead to multiple organ dysfunction and, in some cases, death. As such, early intervention is crucial in reducing mortality and minimizing complications in patients with SAP. Research into the relationship between the microbiota and the pancreas is expanding. Studies show that the oral and rectal microbiota in patients with SAP differ from those in healthy individuals, and their intestinal mucosal barrier is significantly impaired. The extent of this damage correlates with the number of intestinal microbiota and the levels of their metabolic byproducts. Beta diversity of the intestinal microbiota is lower in patients who succumb to SAP [1, 2]. The intestinal microbiota influences the progression of the disease through its metabolic products, such as short-chain fatty acids [3]. Inflammation can increase intestinal mucosal permeability, leading to inflammation of the pancreas and other organs, exacerbating the systemic inflammatory response. This may be linked to systemic inflammatory response syndrome (SIRS) in cases of SAP.

Fecal microbiota transplantation (FMT) is a method to directly change the intestinal microbiota to normalize the composition and gain a therapeutic benefit. Current evidence deems FMT as a generally safe therapeutic method with few adverse effects. The most effective and well-studied indication for FMT is recurrent Clostridium difficile infection. A case report has demonstrated that FMT was an effective initial therapy for pancreatitis complicated with severe Clostridium difficile infection. However, to date, there are few cohort reports about exploring the application of FMT in the treatment of SAP.

Animal studies have shown that Bifidobacterium can help prevent the progression of acute pancreatitis (AP) by regulating pancreatic and systemic inflammatory responses. However, there is limited research on the relationship between changes in gut microbiota and systemic inflammation in patients with SAP. Our study aims to explore whether modifying the gut microbiota in these patients can reduce their systemic inflammation. The primary objective is to lower systemic inflammatory response, reduce mortality, and decrease the incidence of complications by adjusting the gut microbiota in patients with SAP.

## Materials and methods

Data from 28 patients with SAP treated at the First People’s Hospital of Nanning between July 2022 and December 2023 were retrospectively analyzed. Inclusion criteria: (1) Meeting the diagnostic criteria outlined in the Chinese Guidelines for the Diagnosis and Treatment of SAP (2021); (2) Aged between 18 and 60 years; (3) APACHE II score >8, CT Balthazar grading of D or E, or MCTSI grading of at least level II; (4) Signed informed consent from the patient or their family, demonstrating good compliance. Exclusion criteria: (1) Inability to tolerate nasoenteric tube placement; (2) Presence of severe infectious diseases; (3) Conditions, such as intestinal obstruction, gastrointestinal perforation, or acute appendicitis; (4) Malignant tumors; (5) Hospital admission occurring more than 72 h after the onset of symptoms; (6) Pregnant or lactating patients; (7) Prior treatment for AP (e.g., antibiotics) before enrollment. Among the included patients, 13 received standard treatment supplemented with a probiotic capsule containing Bifidobacterium triple viable bacteria (control group). The remaining 15 patients received FMT in addition to standard treatment (FMT group): (1) An intestinal obstruction catheter was placed under gastroscopy within 24 h of enrollment; (2) For three consecutive days, 250 mL of microbial liquid was administered via the nasojejunal tube each day. Additionally, 19 healthy volunteers undergoing routine physical examinations were selected to form the healthy control (HC) group.

### Sample preparation

Before and after treatment, blood samples were taken in the morning to measure clinical indicators, such as complete blood count, C-reactive protein (CRP), procalcitonin (PCT), arterial blood gas analysis, lipase (LPS), amylase, liver function, and kidney function in both the FMT and control group using Mindray BC-7500 and Beckman Coulter AU680 analyzer. Within 24 h of admission, patients in the FMT group and control group were assessed using the APACHE II score, and underwent pancreatic CT scans. Fecal samples were collected from all participants for 16 s rDNA sequencing. The bacterial solution used for FMT was selected according to the “Chinese Expert Consensus on the Selection and Management of Gut Microbiota Transplantation Donors (2022 Edition)”, and the donor was separated according to the “Chinese Expert Consensus on the Establishment and Clinical Application of Standardized Methodology for Microbial Transplantation”. Then, based on the “Chinese Expert Consensus on the Selection and Clinical Application of Microbial Transplantation Pathways”, the upper digestive pathway was selected for transplantation to the patient. In this study, all patients were treated with feces prepared from the same donor to exclude multifactorial confounding caused by abnormal gut microbiota. The dose for a single transplant is 50 g, prepared in a 1:4 ratio with 0.9% physiological saline. The amount of each transplant is 200 mL, and the viable cell count is 2.56 × 10^11^ CFU. Transplant once a day for a total of 4 days.

### The 16s-rDNA sequencing and analysis

The 16s-rDNA sequencing involved fecal DNA extraction, polymerase chain reaction amplification, Illumina high-throughput sequencing, and clustering of operational taxonomic units (OTUs) with 97% consistency. Species annotation was performed on the sequences of the OTUs to obtain corresponding information and abundance distribution.

In community ecology, microbial diversity can be reflected by analyzing the Alpha diversity. Shannon evenness is an index that measures species diversity in a community. The degree of difference in species abundance distribution can be quantitatively analyzed by statistical distance calculation. Principal component analysis (PCA), non-metric multidimensional scaling (NMDS) and principal-coordinate analysis (PCoA) analysis use variance decomposition to reflect Beta diversity from multiple groups.

Venn diagrams can be used to count the number of shared and unique species, intuitively showing the similarity and overlap of composition. Analysis of variance (ANOVA) is used to test the significance of the difference among groups. Receiver operating characteristic curve (ROC curve) with the area under the curve (AUC) was performed to evaluate the diagnostic value of species. The closer the ROC curve is to the upper left corner, the higher the diagnostic accuracy. Additionally, the comparison of different tests can be made by calculating the AUC for each test. The test with the largest AUC has the best diagnostic value. The PICRUSt2 software was used for the OTU sequence, and the IMG microbial genome data was used for KEGG and GO functional information to infer the functional gene composition, and the metabolic pathway histogram was drawn.

### Ethical statement

This study was reviewed and approved by the Ethics Committee of the first people’s hospital of Nanning (No. 2022-071-01) and GuangXi Health Science College.

### Statistical analysis

The data plotting was performed using GraphPad Prism 9, and data statistical analysis was conducted using SPSS 26.0 software. The comparison of qualitative data was analyzed using the chi-square test, while the *t*-test was used for quantitative data. For data that did not follow a normal distribution, the non-parametric Mann–Whitney U test was employed. A *P* value less than 0.05 was considered statistically significant.

## Results

### Clinical characteristics

As shown in Table 1, the proportion of hypertensive patients, APACHE II scores, and the proportion of patients with ≥2 organ dysfunctions were higher in the FMT group when compared to the control group (*P* < 0.05). All SAP patients in this study survived, and patients who received FMT had good treatment outcomes without any observed side effects.

**Table 1:** General characteristics of the enrolled population

**Group**	**Healthy group** **(*n* ═ 19)**	**Control group** **(*n* ═ 13)**	**FMT group** **(*n* ═ 15)**
Male, *n* (%)	4 (21.05)	9 (69.23)	10 (66.67) ^*^
Age, years	46.82 ± 10.75	45.08 ± 10.28	48.87 ± 14.58
Weight, kg	57 ± 9.97	65.13 ± 12.96	84.5 ± 52.54^*^
BMI, kg/m^2^	21.86 ± 2.40	24.09 ± 3.88	29.27 ± 15.12
History of alcohol abuse, *n* (%)		7 (53.85)	5 (33.33)
Combined cholelithiasis, *n* (%)		1 (7.69)	2 (13.33)
Hypertension, *n* (%)		1 (7.69)	6 (40.0)^#^
Diabetes, *n* (%)		2 (15.38)	1 (6.67)
Complications, *n* (%)		12 (92.31)	8 (53.33)
CT grading IV, *n* (%)		13 (100)	14 (93.33)
APACHE II score (points)		25.31 ± 5.33	37.27 ± 12.89^##^
Number of organ disorders ≥ 2, *n* (%)		2 (15.38)	13 (86.67)^###^

The parentheses in the table indicate percentages for different clinical indicator values; Comparison of three groups, *P < 0.05; Comparison between two groups, *^#^P* < 0.05, ^##^*P* < 0.01, ^###^*P* < 0.001. FMT: Fecalmicrobiota transplantation.

### Clinical efficacy comparison

The interleukin-6 (IL-6) and low density lipoprotein (LDL) levels, as well as the number of organ disorders in FMT group patients were significantly higher than those in control group before treatment (*P* < 0.05). Traditional therapy could significantly decrease the levels of red blood cells (RBC), neutrophils (NE), albumin (ALB) and LPS (*P* < 0.05). Comparison of FMT group before and after treatment revealed that FMT could significantly decrease the levels of NE, CRP, PCT, LDL, triglyceride (TG), total cholesterol (TC), lactate dehydrogenase (LDH), LPS and significantly increase calcium (Ca), PH. After treatment, the levels of white blood cell (WBC), NE, CRP, PCT, bilirubin (BIL), creatinine (Cr), D-dimer, LDH, LPS and number of organ disorders significantly decreased and platelet (PLT), ALB, Ca, base excess (BE), PaO_2_ and PH significantly increased in FMT group when compared with control group. As shown in Table 2.

### The abundance and diversity of microbial communities

**Figure 1.**
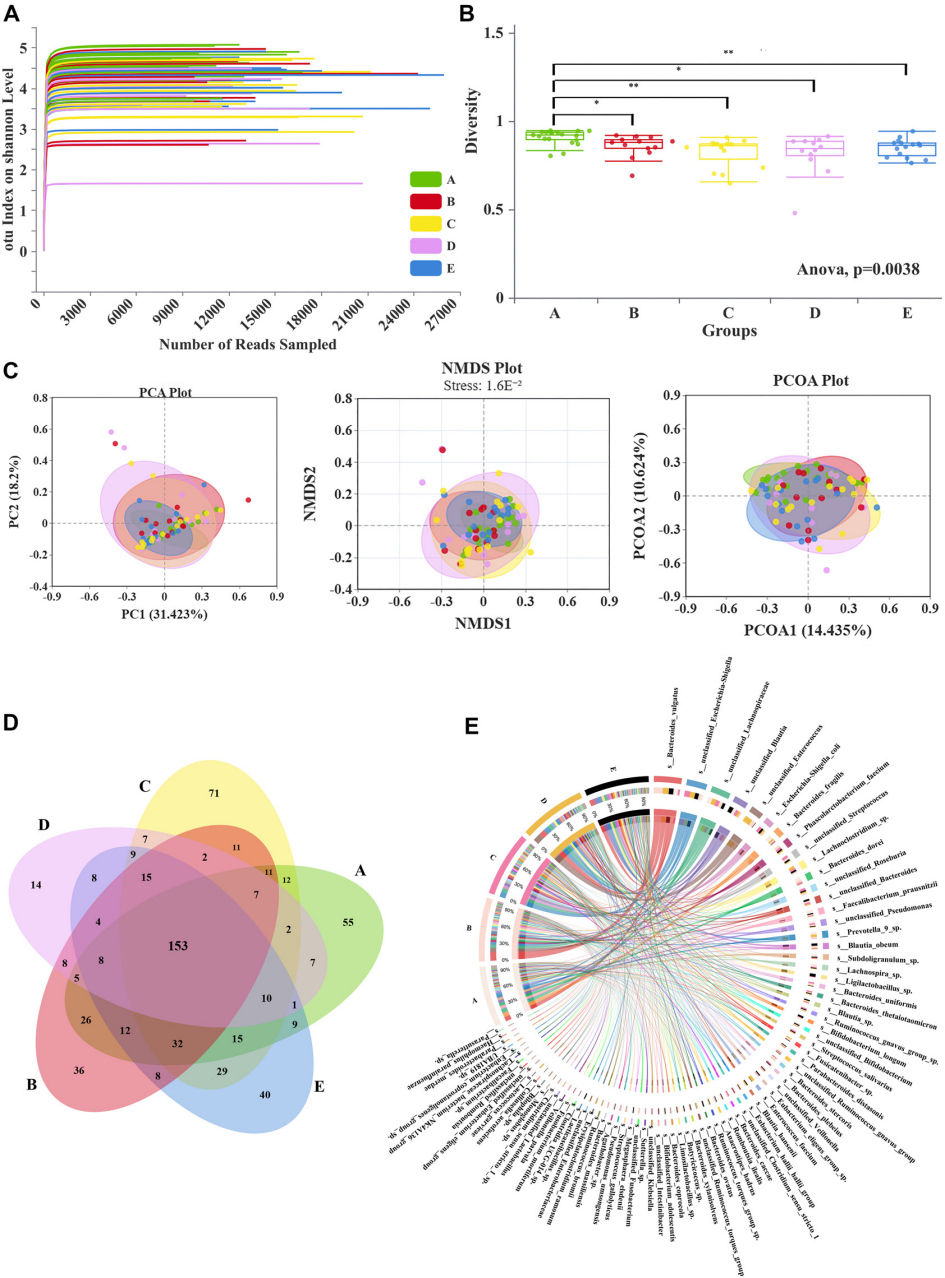
**Sample diversity analysis and species composition.** (A) Dilution curve based on OTU abundance. Randomly selected a certain number of sequences from the sample, calculated the Alpha diversity index of these sequences corresponding to the sample, drawed a curve with the extracted data volume as the horizontal axis and the Alpha diversity index value as the vertical axis, and judged whether the sequencing data volume was sufficient based on whether the curve reaches a plateau. (B) Alpha diversity analysis among groups using shannoneven index (ANOVA method, *P < 0.05, **P < 0.01). The axis represented the group name, and the vertical axis represented the Alpha index of each group. (C) Beta diversity analysis using PCA, NMDS, and PCoA methods. Each point was a sample, and the closer the two sample points are, the more similar the species composition of the two samples. The horizontal and vertical axes represented relative distances and had no practical significance. Stress: Evaluate the quality of NMDS analysis results. When stress < 0.05, it had good representativeness. 1.6E-2 ═ 0.016. (D) Venn diagram: Overlapping numbers represented the number of species shared among multiple groups, and non overlapping numbers represented the number of species unique to the corresponding group. There were 153 common microbial species among 5 groups. (E) Circos diagram: The dominant microbial species included *Bacteroides vulgatus*, *Escherichia Shigella*, *Lachnospiraceae*, and so on. A: Healthy group; B: Control group before treatment; C: FMT group before treatment; D: Control group after treatment; E: FMT group after treatment. FMT: Fecal microbiota transplantation; ANOVA: Analysis of variance; PCA: Principal component analysis; PCoA: Principal-coordinate analysis; NMDS: Non-metric multidimensional scaling; OTU: Operational taxonomic unit.

The dilution curves (Figure 1A) for each sample are relatively flat, indicating that the sequencing depth has sufficiently captured all species and that the sampling strategy is generally appropriate. This allows for a more accurate reflection of species diversity within the samples. Alpha diversity provides insights into the abundance and diversity of microbial communities. When comparing the community richness indices, specifically ShannonEven, a significant difference was observed among the three groups (Figure 1B, *P* ═ 0.0038). Differences in species abundance distributions can be quantitatively assessed using statistical distances. Beta diversity analysis, which visualizes and examines the relationships among groups, revealed distinct microbiome compositions across groups. The Beta diversity analyses (Figure 1C), including PCA, NMDS, and PCoA, demonstrated clear group separations (Stress < 0.05).

### Species composition analysis

**Figure 2.**
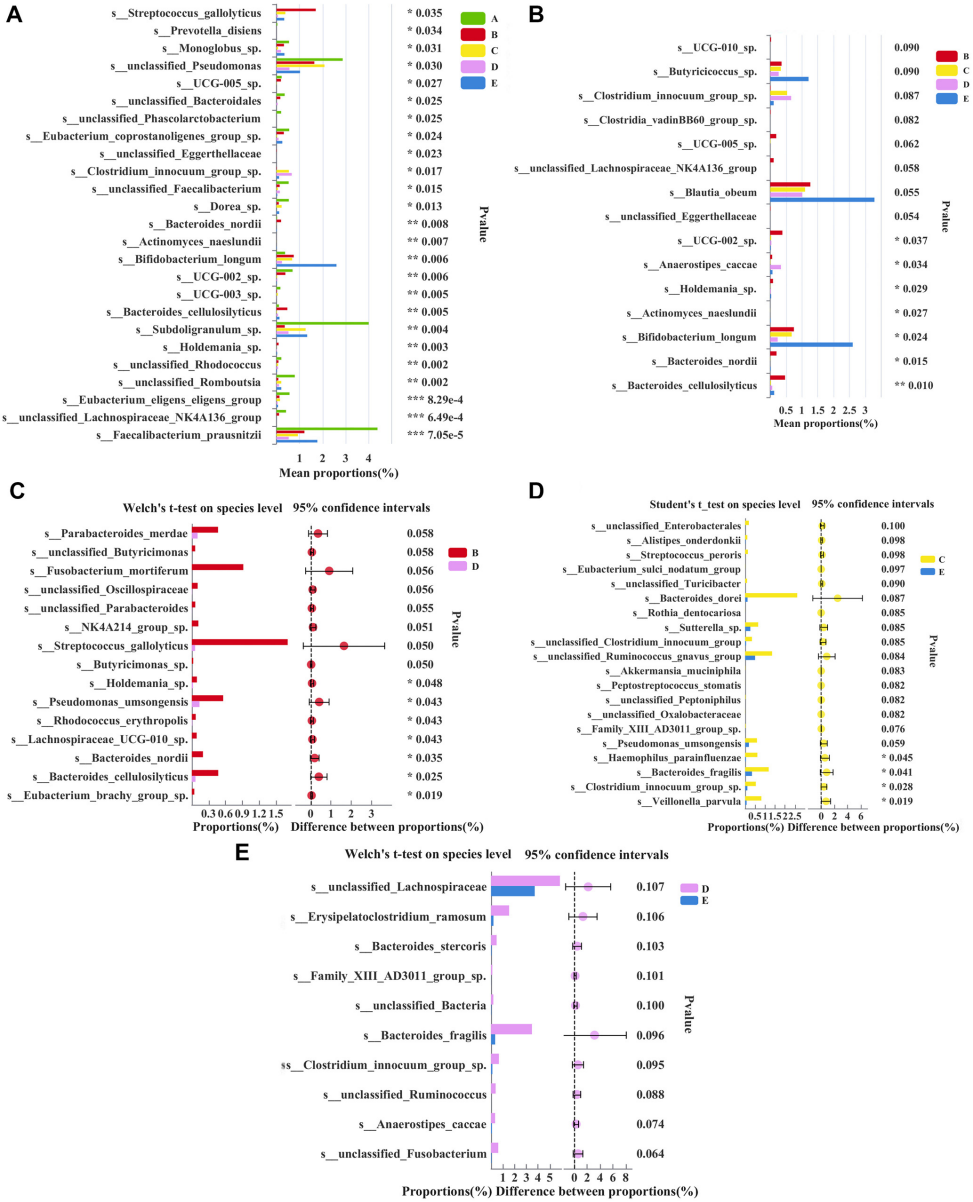
**Species difference analysis.** (A) Relative abundance of microbial species among five groups; (B) Relative abundance of microbial species among BCDE groups; (C) Relative abundance of microbial species between BD groups; (D) Relative abundance of microbial species between CE groups; (E) Relative abundance of microbial species between DE groups. A: Healthy group; B: Control group before treatment; C: FMT group before treatment; D: Control group after treatment; E: FMT Group after treatment (*P ≤ 0.05, ** *P* ≤ 0.01, *** *P* ≤ 0.001). FMT: Fecal microbiota transplantation.

At the species level, 153 common microbial species were identified across groups (Figure 1D). The dominant microbial species include *Bacteroides vulgatus*, *Escherichia-Shigella*, *Lachnospiraceae*, and others (Figure 1E). A comparison among the five groups is shown in Figure 2A. Notably, *Pseudomonas*, *Subdoligranulum*, and *Lachnospiraceae* NK4A136 were significantly decreased in patients with SAP (*P* < 0.05), whereas *Bifidobacterium longum* significantly increased in the FMT group after treatment (Figure 2B, *P* < 0.05). In the control group, comparisons before and after treatment revealed significant decreases in *Holdemania*, *Pseudomonas umsongensis*, *Rhodococcus erythropolis*, *Lachnospiraceae* UCG-010, *Bacteroides nordii*, Bacteroides *cellulosilyticus*, and *Eubacterium brachy* in patients with SAP after traditional treatment (Figure 2C, *P* < 0.05). Meanwhile, in the FMT group, comparisons before and after treatment showed that FMT significantly decreased the levels of *Veillonella parvula*, *Clostridium innocuum*, *Bacteroides fragilis*, and *Haemophilus parainfluenzae* in patients with SAP (Figure 2D, *P* < 0.05).

### ROC curve analysis

**Figure 3.**
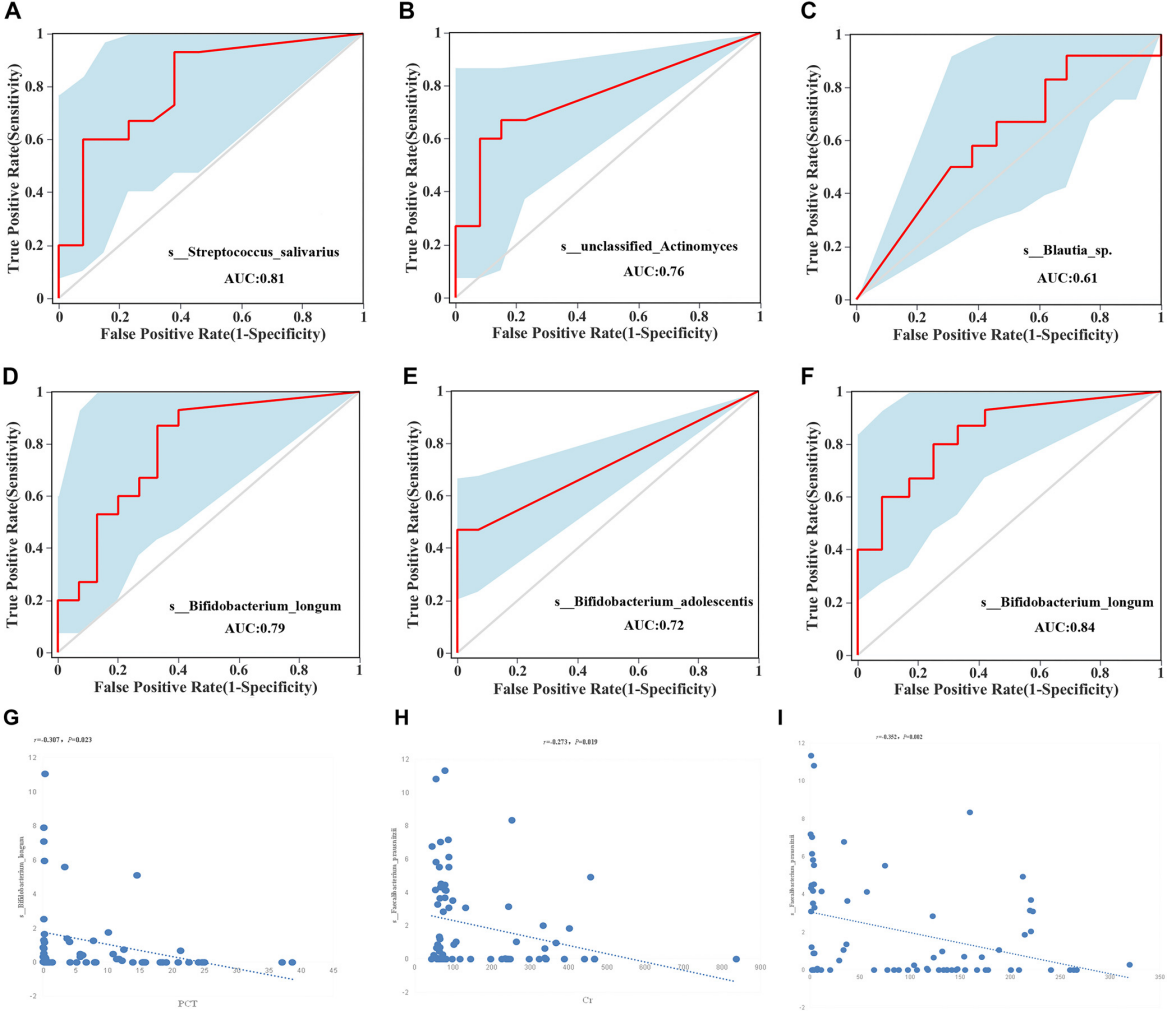
**ROC curve.** Plot the ROC curve with sensitivity as the vertical axis representing true positive rate and specificity as the horizontal axis representing false positive rate. All *P* < 0.05. Before treatment, *Streptococcus salivarius* (A, AUC ═ 0.81) and *Actinomyces* (B, AUC ═ 0.76) could be regard as distinguishing species among healthy group, control group and FMT group. (C) Distinguishing species for control group before and after treatment was *Blautia* (AUC ═ 0.61). Distinguishing species for FMT group before and after treatment was *Bifidobacterium longum* (D, AUC ═ 0.79) and *Bifidobacterium adolescentis* (E, AUC ═ 0.72). (F) Distinguishing species for control group and FMT group after treatment included *Bifidobacterium longum* (F, AUC ═ 0.84). The abundance of *Clostridium perfringens* was positively correlated with blood Ca and CRP values (G and H), and negatively correlated with Cr values (I). FMT: Fecal microbiota transplantation; Ca: Calcium; Cr: Creatinine; CRP: C-reactive protein; AUC: Area under the curve; ROC: Receiver operating characteristic.

Before treatment, *Streptococcus salivarius* (Figure 3A, AUC ═ 0.81) and *Actinomyces* (Figure 3B, AUC ═ 0.76) could be regard as distinguishing species among healthy group, control group and FMT group. Distinguishing species for control group before and after treatment included *Blautia* (Figure 3C, AUC ═ 0.61). Distinguishing species for FMT group before and after treatment included *B. longum* (Figure 3D, AUC ═ 0.79) and *Bifidobacterium adolescentis* (Figure 3E, AUC ═ 0.72). Distinguishing species for control group and FMT group after treatment included *B. longum* (Figure 3F, AUC ═ 0.84). The relative abundance of *B. longum* was negatively correlated with PCT values (Figure 3G, *P* ═ 0.023), while the abundance of *Clostridium perfringens* was positively correlated with blood Ca and CRP values (Figure 3H and 3I, *P* ═ 0.01 and 0.002), and negatively correlated with Cr values (Figure 3J, *P* ═ 0.019).

## Discussion

Our findings showed that the FMT group had greater improvements in clinical indicators related to the severity of AP, including CRP, PCT, serum Ca, and APACHE II scores, compared to the control group. The incidence and mortality rates of multiple organ dysfunction were also lower in the FMT group than in the control group.

Significant differences in gut microbiota diversity and dominant bacterial species were observed between the AP group and the HC group, with the HC group having significantly higher levels of *Subdoligranulum* and *Faecalibacterium prausnitzii*. In the AP group, there were notable changes in gut microbiota composition following FMT and standard treatment. The abundance of *B. longum* was lower in the AP group treated with the *Bifidobacterium*, but increased significantly after FMT treatment compared to the control group. The predicted AUC value for this outcome was 0.84.

Based on these data, we conducted a correlation analysis between clinical indicators—such as CRP, PCT, serum Ca, and CT—and gut microbiota. The results showed that PCT levels were negatively correlated with Bifidobacterium longum abundance (*P* ═ 0.023), and serum Ca and Cr were negatively correlated with *Faecalibacterium prausnitzii* abundance (*P* ═ 0.02 and *P* ═ 0.019, respectively).

**Table 2:** Comparison of FMT group and oral tablet treatment group before and after treatment

**Clinical index**	**Control group (*n* ═ 13)**		**FMT group (*n* ═ 15)**	
	**Before treatment**	**After treatment**	**Before treatment**	**After treatment**
RBC, ×10^12^/L	4.85 ± 1.29	3.67 ± 1.24^c^	3.94 ± 1.54	3.86 ± 0.93
WBC, ×10^9^/L	15.92 ± 2.69	14.27 ± 3	14.87 ± 4.05	8.05 ± 2.1^bbbb^
LYMP, ×10^9^/L	1.15 ± 0.43	1.24 ± 0.34	0.96 ± 0.35	1.32 ± 0.61
NE, ×10^9^/L	14.28 ± 2.69	11.54 ± 3.61^c^	12.58 ± 4.3	5.91 ± 1.73^bbbdddd^
CRP, mg/L	169.41 ± 37.5	164.88 ± 54.57	147.26 ± 89.91	45.13 ± 35.97^bbbbdd^
PCT, ng/mL	13.98 ± 6.56	14.83 ± 8.89	8.34 ± 10.13	0.23 ± 0.22^bbbd^
IL-6, pg/mL	19.76 ± 5.61	22.12 ± 4.56	13.76 ± 5.96^a^	13.06 ± 14.82
PLT, ×10^9^/L	195.54 ± 74.8	191.54 ± 106.72	255.6 ± 121.46	333.07 ± 93.23^bb^
ALB, g/L	34.87 ± 3.84	30.75 ± 2.48^cc^	35.12 ± 6.39	35.55 ± 5.29^b^
BIL, µmol/L	29.65 ± 9.82	29.99 ± 14.72	26.76 ± 32.34	11.69 ± 6.42^bb^
TG, mmol/L	9.22 ± 6.58	5.05 ± 2.07	8.33 ± 5.83	3.86 ± 1.05^d^
HDL, mmol/L	1.53 ± 0.72	1.45 ± 0.5	1.2 ± 0.93	1.69 ± 0.87
LDL, mmol/L	2.33 ± 1.15	2.41 ± 0.8	2.01 ± 1.32	1.93 ± 0.5
TC, mmol/L	5.36 ± 1.83	4.48 ± 0.39	7.23 ± 2.82	7.23 ± 2.82^dd^
Cr, µmol/L	239.62 ± 118.21	242.69 ± 83.61	229.19 ± 221.87	101 ± 96.67^bbb^
BUN, mmol/L	10.32 ± 3.72	9.7 ± 1.23	10.36 ± 9.15	6.82 ± 9.41
Ca, mmol/L	1.9 ± 0.41	1.8 ± 0.28	1.67 ± 0.49	2.3 ± 0.46^bbdd^
GLU, mmol/L	13.84 ± 7.05	10.81 ± 2.54	11.1 ± 5.65	9.14 ± 3.95
D-dimer, mg/L	5.07 ± 1.86	6.65 ± 3.11	8.2 ± 8.31	3.53 ± 1.23^bb^
BE, mmol/L	−8.6 ± 5.9	−5.58 ± 3.52	−3.03 ± 8.14	0.43 ± 2.84^bbbb^
LDH, U/L	461 ± 164.02	500.92 ± 481.75	304.83 ± 79.91^aa^	186.18 ± 54.23^bdd^
PaO_2_, mmHg	80.98 ± 26.74	81.82 ± 7.51	94.21 ± 25.72	98.85 ± 27.29^b^
PH	7.37 ± 0.06	7.39 ± 0.03	7.36 ± 0.09	7.43 ± 0.05^bd^
Amy, U/L	1660.08 ± 2803.48	207.92 ± 75.36	1724.73 ± 2796.98	87.47 ± 53.31^bbbb^
LPS, U/L	1065 ± 751.25	219.46 ± 38.58^cc^	1784.04 ± 1530.29	98.25 ± 94.3^bbbdd^
Number of organ disorders ≥ 2	2	7	13^aaa^	1^b^
Survival	13	11	15	14

FMT: Fecal microbiota transplantation; RBC: Red blood cell; WBC: White blood cell; LYMP: Lymphocyte; NE: Neutrophils; CRP: C-reactive protein; PCT: Procalcitonin; IL-6: Interleukin-6; PLT: Platelet; ALB: Albumin; BIL: Bilirubin; TG: Triglyceride; HDL: High density lipoprotein; LDL: Low density lipoprotein; TC: Total cholesterol; Cr: Creatinine; BUN: (Urea nitrogen), Ca (Blood calcium), GLU (Blood sugar); BE: Base excess; LDH: Lactate dehydrogenase; PaO_2_: Arterial partial pressure of oxygen; PH: PH value; Amy: Amylase; LPS: Lipase. a: Comparison between the FMT group and the control group before treatment; b: Comparison between the FMT group and the control group after treatment; c: Comparison between the control group before and after treatment; d: Comparison of FMT group before and after treatment.

SAP is a serious inflammation of the pancreas and associated with severe morbidity and mortality [1]. The changes in the intestinal microbiota structure are involved in the process of pancreatitis and its complications. Related studies have shown that probiotics, especially mixed probiotics, can reduce systemic inflammatory response caused by AP by reducing inflammation and protecting intestinal mucosal permeability [4]. Many studies have reported that FMT is a safe and effective treatment that can restore the balance of the intestinal microbiota., mainly aimed at intestinal dysfunction or dysfunction caused by *Clostridium* infection, chronic enteritis, irritable bowel syndrome, ulcerative colitis, Crohn’s disease, and other etiologies [5, 6]. However, the therapeutic efficacy and underlying mechanisms of FMT in SAP remain to be elucidated. In this study, we carried out a cohort research of 19 healthy individuals and 28 SAP patients (13 in control group and 15 in FMT group) to explore the efficacy and underlying mechanisms of FMT in SAP.

Hypertension is closely related to the occurrence and development of pancreatitis, and even pancreatic dysfunction can occur due to changes caused by arterial sclerosis induced by hypertension [7]. In this study, the proportion of patients with hypertension was higher in the FMT group than in the control group. APACHE II is currently the most widely used prognostic scoring system for SAP and an important indicator for distinguishing mild and severe SAP. An APACHE II score of >═ 8 is considered as SAP. Based on the APACHE II score, patients in the FMT group had a higher severity compared to the control group before treatment.

The levels of WBC, NE, CRP, PCT, LPS significantly decreased in FMT group when compared with control group after treatment. These findings indicate that FMT treatment can effectively reduce systemic inflammation in patients with pancreatitis. In addition, a global systematic review has shown that hypertriglyceridemia is a cause of SAP [8]. In our research, FMT group showed a significant decrease in TG levels after treatment. Therefore, we believed that FMT may play a role in reducing TG levels and effectively control the impact of TGs on the patient’s condition. Studies have shown that more SAP is associated with lower PLT counts and higher D-dimer levels [9]. Both groups had elevated D-dimer levels before treatment, and the FMT group had significantly lower levels than the control group after treatment, indicating that FMT can significantly reduce the harm of SAP on the coagulation and fibrinolysis systems.

Studies have shown that LDH is related to the severity of the disease [10]. In our research, LDH levels significantly decreased after FMT treatment. Therefore, we believe that FMT may alleviate the condition of patients by reducing LDH levels. The diagnosis of SAP includes serum amylase or lipase levels that are at least three times above the upper limit of normal [11]. During the course of SAP, abnormal secretion of pancreatic proteases and destruction of pancreatic structure may lead to gut imbalance and alterations in the intestinal microbiota [12, 13]. Both groups showed no significant differences in amylase or lipase levels before treatment. However, after treatment, these levels significantly decreased, with the FMT group showing lower levels than the control group. Additionally, the proportion of patients with two or more organ dysfunctions significantly increased in the control group after treatment, while it significantly decreased in the FMT group. This suggests that FMT can effectively control the severity of SAP.

In clinical practice, PLTs play a crucial role in promoting blood clotting and hemostasis. In this study, the FMT group showed higher PLT levels than the control group after treatment, indicating that FMT may not affect the coagulation and hemostasis functions of PLTs. Research has shown that the most common cause of low blood Ca is low serum ALB [14]. After treatment, the control group showed a significant decrease in ALB levels, which were significantly lower than those in the FMT group. The Ca levels increased significantly in FMT group after treatment. This suggests that FMT treatment can improve low ALB and low blood Ca levels in patients. Elevated Cr levels are closely related to the occurrence of pancreatic necrosis in patients with SAP [15]. There was no significant difference in Cr levels between the control and FMT groups before treatment, but after treatment, the FMT group had lower levels than the control group. This indicates that FMT can effectively prevent pancreatic necrosis in patients, also prevent early acute kidney injury [16]. BE is an independent risk factor for 60-day mortality in patients with pancreatitis [17]. Both groups had decreased BE levels before treatment, with no significant difference between them. After treatment, the FMT group returned to normal levels. Accordingly, the FMT group showed better treatment outcomes and improved relief of symptoms compared to the control group.

We explored the mechanism of FMT therapy through 16 s RNA sequencing, Alpha diversity of the intestinal microbiota in the SAP patients (including before and after treatment) was lower when compared with the healthy group. Many studies have reported that α-diversity was down-regulated by various diseases, such as overweight, colorectal cancer, neurological and psychiatric disorders [18, 19]. Beta diversity showed that the microbiome composition among groups was obviously distinctive. We compared the four groups of pancreatitis, including the control group before and after treatment, and the FMT group before and after treatment. We found that *Bifidobacterium longum* significantly increased in the FMT group after treatment. *Bifidobacterium longum* is a probiotic and a gram-positive, non-spore-forming bacterium that is an important member of the human intestinal microbiota [20]. Bifidobacterium longum naturally exists in the human gastrointestinal tract and is one of the earliest bacteria to colonize the intestine during birth [21]. It has various physiological functions, such as lowering intestinal pH, inhibiting the growth of pathogens, enhancing immune function, and promoting nutrient absorption [22]. It can produce lactic acid and other organic acids through fermentation to maintain intestinal acid-base balance and inhibit the growth of harmful bacteria. In addition, *Bifidobacterium longum* can produce antibacterial substances, such as bacteriocins and organic acids, which help maintain the balance of the intestinal microbiota [23]. *Bifidobacterium longum* is also acid- and bile-tolerant and can adapt to the intestinal environment, colonizing and persisting in the gut. Therefore, *Bifidobacterium longum* is commonly used in the production of probiotic beverages and health products to help maintain intestinal health [24]. According to relevant literature, *Escherichia coli*, *Enterococcus*, and *Enterobacteriaceae* are the main pathogens of secondary intestinal infections caused by AP. Researchers examined the classification of gut microbiota in SAP patients and found an imbalance in proportion, characterized by an increase in the proportion of aerobic bacteria, mainly *Escherichia coli*, while the growth of anaerobic bacteria such as *Bifidobacterium* was inhibited, resulting in a decrease in the number of these bacteria. In the human gut, probiotics compete with harmful bacteria to inhibit the growth of pathogens. In addition, the bioactive substances produced by probiotics can inhibit or kill pathogenic bacteria, thereby regulating the balance of gut microbiota. Probiotics can also stimulate the secretion of mucin by the intestinal mucosa, inhibit the binding of pathogenic bacteria to the intestinal epithelium, reduce their passage through the intestinal wall into peripheral organs, promote the biosynthesis of glutathione, improve intestinal mucosal barrier dysfunction, reduce oxidative stress, decrease ileal mucosal permeability and cell apoptosis levels, thereby inhibiting intestinal bacterial migration and affecting the function of immune cells (for example, by regulating the production of pro-inflammatory and anti-inflammatory cytokines) [25]. In our study, before treatment, the levels of *Bifidobacterium longum* and *Faecalibacterium prausnitzii* in both the FMT group and the control group were significantly lower than those in the healthy population group. After treatment, the levels of Bifidobacterium longum and *Faecalibacterium prausnitzii* in the FMT group increased, with significant differences observed before and after treatment. The increase in these two groups of bacteria in the control group was not significant, and there was no difference in the comparison before and after, indicating that the increase in these two types of bacteria is related to FMT. We believe that SAP patients do have a decrease in bifidobacteria, which is likely related to intestinal damage and increased oxidative stress. Through FMT treatment, *Bifidobacterium* significantly increased and the number of organs damaged significantly decreased, showing improvement in patient prognosis. This indicates that FMT can indeed increase beneficial gut microbiota, thereby inhibiting the growth of harmful microbiota, regulating gut microbiota, improving gut function, reducing inflammatory response, and promoting prognosis.

*Bifidobacterium longum* can distinguish both the pre-treatment FMT group and the post-treatment FMT group, as well as the post-treatment FMT group and the post-treatment control group, with much higher abundance observed in the post-treatment FMT group compared to the pre-treatment FMT group and the post-treatment control group, indicating its important role in FMT therapy. The functional abundance analysis of the detected bacteria showed relative functions, such as chemoheterotrophy, fermentation, animal parasites or symbionts, ATP-binding cassette subfamily B, etc., which highly possibly support the treatment of pancreatitis.

Nonetheless, our study also has some limitations. Firstly, our research sample size is still not large enough. Due to the fact that we are only conducting exploratory experiments in the early stage, the number of research subjects we have included is not sufficient, and the universal applicability of the research results is still insufficient. In addition, we must consider the impact of gene polymorphism on research results, which we have not yet analyzed in our current research. In the future, we can further validate our research results through multi center and diverse clinical trials, and incorporate multiple factors such as genes into the research analysis.

## Conclusion

In summary, FMT therapy for pancreatitis has a significant effect and can effectively improve various clinical indicators. The increase in abundance of Bifidobacterium longum in the intestinal microbiota may be the reason for the significant therapeutic effect of FMT therapy.

## Data Availability

Data are available from the corresponding authors.
